# Supplementary material for: Hybrid Models and Biological Model Reduction with PyDSTool
Source: PLoS Comput Biol. 2012 Aug 9;8(8):e1002628. doi: 10.1371/journal.pcbi.1002628 (PMC3415397; doi:10.1371/journal.pcbi.1002628)
Supplement: Text S4 — Complete source code for the PyDSTool package (version 0.88.120504). Includes API documentation and help files linking to web pages. This file is identical to the current public release on Sourceforge.net. (ZIP) [file pcbi.1002628.s004.zip › PyDSTool/html/PyDSTool.PyCont.Continuation.HopfCurveTwo-class.html]

xml version="1.0" encoding="ascii"?


PyDSTool.PyCont.Continuation.HopfCurveTwo


| Home | Trees | Indices | Help | PyDSTool |
| --- | --- | --- | --- | --- |

Package PyDSTool ::
Package PyCont ::
Module Continuation ::
Class HopfCurveTwo

[hide private]

[frames] | no frames]

# Class HopfCurveTwo

source code

```
  object --+    
           |    
Continuation --+
               |
              HopfCurveTwo
```

---

```
Child of Continuation class that represents curves of Hopf points.

Augmented system h(x,a): Uses double bordering on the matrix A given by

        A = f_{x}^{2}(x,a) + _k*I_{n}

    (see class PyCont.TestFunc.BorderMethod)
    (see class PyCont.TestFunc.Hopf_Double_Bor_Two)

    The continuation variables are (x,a,_k) with

        x = State variables
        a = Free parameters
        _k = w^2 = Square of pure imaginary eigenvalue associated with Hopf point
            (auxiliary parameter)

    and the continuation curve is defined by

                    { f(x,a) = 0
        h(x,a,_k) = { G_{1,1}(x,a,_k) = 0
                    { G_{2,2}(x,a,_k) = 0

Detection of points: BT, ZH, GH
```


|  |  |
| --- | --- |
| Instance Methods | [hide private] |

|  |  |
| --- | --- |
| \_\_init\_\_(self, model, gen, automod, plot, args=None)  x.\_\_init\_\_(...) initializes x; see x.\_\_class\_\_.\_\_doc\_\_ for signature | source code |

|  |  |
| --- | --- |
| update(self, args)  Update parameters for HopfCurveTwo. | source code |

|  |  |
| --- | --- |
| \_createTestFuncs(self)  Creates processors and test functions for HopfCurveTwo class. | source code |

**Inherited from `Continuation`**:
`__copy__`,
`__deepcopy__`,
`__repr__`,
`__str__`,
`backward`,
`cleanLabels`,
`computeEigen`,
`display`,
`exportGeomview`,
`forward`,
`getSpecialPoint`,
`info`,
`reset`,
`testdomain`,
`testdomaingrid`,
`testdomaintangrid`

**Inherited from `Continuation`** (private):
`_Keller`,
`_MoorePenrose`,
`_Natural`,
`_checkForBifPoints`,
`_compute`,
`_curveToPointset`,
`_preTestFunc`,
`_savePointInfo`,
`_system`,
`_systemjac`,
`_systemjac_withpars`,
`_systemjacuser`,
`_systemuser`

**Inherited from `object`**:
`__delattr__`,
`__getattribute__`,
`__hash__`,
`__new__`,
`__reduce__`,
`__reduce_ex__`,
`__setattr__`


|  |  |
| --- | --- |
| Properties | [hide private] |

**Inherited from `object`**:
`__class__`


|  |  |
| --- | --- |
| Method Details | [hide private] |

|  |  |
| --- | --- |
| \_\_init\_\_(self, model, gen, automod, plot, args=None)  *(Constructor)* | source code |

x.\_\_init\_\_(...) initializes x; see x.\_\_class\_\_.\_\_doc\_\_ for
signature

Overrides: object.\_\_init\_\_: *(inherited documentation)*

|  |  |
| --- | --- |
| update(self, args) | source code |

Update parameters for HopfCurveTwo.

Overrides: Continuation.update

|  |  |
| --- | --- |
| \_createTestFuncs(self) | source code |

Creates processors and test functions for HopfCurveTwo class.

Note: In the following list, processors are in PyCont.Bifpoint and
test functions are in PyCont.TestFunc.

Point type (Processor): Test function(s)
----------------------------------------------

BT (BTPoint): BT\_Hopf ZH (ZHPoint): Fold\_Det (or Fold\_Bor) GH
(GHPoint): GH\_Hopf

Overrides: Continuation.\_createTestFuncs

  


| Home | Trees | Indices | Help | PyDSTool |
| --- | --- | --- | --- | --- |

|  |  |
| --- | --- |
| Generated by Epydoc 3.0.1 on Fri May 4 15:24:07 2012 | http://epydoc.sourceforge.net |
